# ACE and chitotriosidase as possible predictive biomarkers for activity of sarcoidosis in correlation with PET/CT findings

**DOI:** 10.5937/jomb0-50994

**Published:** 2025-03-21

**Authors:** Mihailo Stjepanovic, Zorica Sumarac, Drazenka Grubac, Slobodan Belic, Nikola Maric, Natasa Djurdjevic, Aleksandar Sumarac, Jelena Jankovic, Snezana Jovicic

**Affiliations:** 1 University Clinical Centre of Serbia, Clinic of Pulmonology, Belgrade; 2 University of Belgrade, Faculty of Medicine, Belgrade; 3 University Clinical Centre of Serbia, Centre for Medical Biochemistry, Belgrade; 4 University of Novi Sad, Faculty of Pharmacy, Novi Sad; 5 Public Health Institution - Hospital Trebinje, Trebinje, Republic of Srpska, Bosnia and Herzegovina; 6 University of Belgrade, Faculty of Pharmacy, Department for Medical Biochemistry, Belgrade

**Keywords:** sarcoidosis, ACE, chitotriosidase, PET/CT, activity, sarkoidoza, ACE, hitotriozidaza, PET/CT, aktivnost

## Abstract

**Background:**

Sarcoidosis is a granulomatous disease which can afflict virtually any tissue in the human body, most commonly the mediastinal lymph nodes and lungs. Pathohistological confirmation is the gold standard in establishing a diagnosis; however, determining the activity of the disease requires multiple clinical, radiographic and laboratory procedures. PET/CT scan is considered the gold standard for determining the presence of active granuloma but has several significant limitations (radioactive material, cost, overall access to device). ACE and chitotriosidase are biomarkers used for diagnosing sarcoidosis and could have a place in determining the activity of the disease when compared with the results of PET/CT scans.

**Methods:**

We have compared the levels of ACE and chitotriosidase with the levels of SUVmax values in patients with sarcoidosis.

**Results:**

SUVmax and chitotriosidase levels were significantly correlated at the baseline and after the follow-up period, regardless of gender, age, duration of disease and radiography stage, while SUVmax and ACE levels were not. Chitotriosidase also showed a significant predictive ability to decrease the activity of sarcoidosis, which represented the decrease of SUVmax as the effect of therapy compared with ACE.

**Conclusions:**

In the absence of an ideal biomarker for sarcoidosis (high sensitivity, specificity and stability), chitotriosidase can be used in determining the activity of the disease, as it has shown a significant correlation to the gold standard-PET/CT scan.

## Abstract (sr)

**Uvod:**

Sarkoidoza je granulomatozna bolest koja može da zahvati praktično svako tkivo u ljudskom telu, najčešće medijastinalne limfne čvorove i pluća. Patohistološka potvrda je zlatni standard u postavljanju dijagnoze, međutim, za određivanje aktivnosti bolesti neophodno je primeniti više kliničkih, radiografskih i laboratorijskih procedura. PET/CT skeniranje se smatra zlatnim standardom za određivanje prisustva aktivnog granuloma, ali ima nekoliko značajnih ograničenja (radioaktivni materijal, cena, dostupnost). ACE i hitotriozidaza su biomarkeri koji se koriste u dijagnostici sarkoidoze i mogu da imaju svoje mesto u određivanju aktivnosti bolesti u poređenju sa rezultatima PET/CT skeniranja.

**Metode:**

Uporedili smo nivoe ACE i hitotriozidaze sa nivoima SUVmax vrednosti kod pacijenata sa sarkoidozom.

**Rezultati:**

SUVmax i nivo hitotriozidaze su pokazali korelaciju kako na početku tako i nakon perioda praćenja, bez obzira na pol, starost, trajanje bolesti i stadijum radiografije, dok SUVmax i nivo ACE nisu. Hitotriozidaza je takođe pokazala značajnu prediktivnu sposobnost smanjenja aktivnosti sarkoidoze predstavljene kao smanjenje SUVmax kao efekta terapije u poređenju sa ACE.

**Zaključak:**

U nedostatku idealnog biomarkera za sarkoidozu (visoka osetljivost, specifičnost i stabilnost), hitotriozidaza se može koristiti za određivanje aktivnosti bolesti, jer je pokazala značajnu korelaciju sa zlatnim standardom - nalazom PET/CT snimanja.

## Introduction

Sarcoidosis is an inflammatory disease characterised by non-caseous granuloma that develops in tissue. The clinical presentation varies depending on the afflicted tissue, but most commonly, patients complain of fatigue, chest pain, difficulty breathing, swelling, and joint pain in the acute phase of the disease. A certain number are asymptomatic. Atypical manifestations and imaging findings can make diagnosis more challenging, in the first place, cardiac sarcoidosis and neurosarcoidosis. It is an unpredictable disease, and we are never sure if it is going to relapse or if it is going to turn into a chronic form, and of course, it can remain asymptomatic. The aggravatingcircumstance is that no single sign, symptom, or diagnostic method shows in which direction the disease will develop. It is difficult to assess disease activity in patients with chronic form, older people and those with multiple comorbidities because the symptoms of sarcoidosis are nonspecific and often mimic the worsening of other diseases, and vice versa.

Procedures used to assess disease activity are anamnestic data, physical examination, chest X-ray, examination of respiratory function, 6-minute walk test, magnetic resonance imaging (targeted affected organ), etc. Each of these diagnostic procedures/laboratory analyses independently does not have high specificity and sensitivity, so clinicians use two or more diagnostic methods in daily practice to increase the value of these two parameters. The most common biomarkers used in current practice are angiotensin-converting enzyme (ACE) in serum or plasma and calcium in 24-hour urine. In recent years, the applicability of other biomarkers such as soluble IL-2 receptor (sIL-2R), Krebs von den lungen-6 (KL-6), chitinase-like cartilage glycoprotein (YKL-40), neopterin, and adenosine deaminase (ADA) has been investigated [1] [2] [3] [4]. Among them, chitotriosidase (CHT) has shown particularly great importance in the diagnosis and therapy monitoring of patients with sarcoidosis [1] [5] [6] [7]. Scientific research dealing with diagnosing and treating patients with sarcoidosis examines the differences in the applicability of ACE and CHT, determining the sensitivity and specificity of markers and mutual correlations and correlations with other diagnostic procedures and clinical signs [5] [6].

Sarcoid granuloma is composed, among other things, of activated macrophages and CD4+ lymphocytes. These cells show the presence of glucose transporters (GLUTs), predominantly GLUT-1 and GLUT-3, in their cell membrane. The mentioned transporters, such as transporting glucose, also carry FDG into lymphocytes and macrophages, and therefore, positron emission tomography/computerised tomography (PET/CT) can be used as a diagnostic tool in this disease [8]. Positron emission tomography-computed tomography (PET/CT) scan is a diagnostic procedurethat is not routinely used. However, it has a high yield in determining the location of active lesions and can help diagnose extrapulmonary locations of sarcoidosis and re-evaluate the efficacy of treatment. Maximum standardised uptake value (SUVmax) is used to determine the disease’s activity, which differs for each tissue. The utility of PET/CT was first published by Lewis and Salama, followed by several other researchers [9]. Teirstein et al. were the first to report that positive pulmonary FDG PET findings occurred in two-thirds of patients with radiographic stage II and III sarcoidosis. Negative pulmonary FDG PET findings were common in patients with radiographic stage 0, I and IV sarcoidosis [10]. This procedure is not the most effective in patients with a chronic form of the disease since the duration of the disease can affect the results. Sarcoidosis is in remission, or stage IV of the disease (fibrosis) on PET/CT shows anatomical tissue damage, but the disease may be inactive at that moment. Based on several studies, it was concluded that PET is the gold standard for active sarcoidosis in newly diagnosed and pathologically verified cases [11]. Thirteen studies using FDG-PET or FDGPET/CT in biopsy-proven sarcoidosis showed sensitivities ranging from 89 to 100% [11].

This study aimed to test whether there is a correlation between the levels of biomarkers in patients with histologically confirmed sarcoidosis and the level of SUVmax on PET/CT and whether ACE or CHT can indicate the change in the activity of the disease.

## Materials and methods

We randomly selected 90 patients with histologically diagnosed sarcoidosis treated in the Clinic of Pulmonology University Clinical Centre of Serbia to participate in this study. The local ethics committee’s approval was obtained before the study started (Ethics Committee of the University Clinical Centre of Serbia). The disease activity was estimated using clinical presentation, radiological findings, biomarkers (ACE and CHT), and PET/CT scans. The same data was collected on follow-up after 6–9 months. Patients included in this study were not taking medications that interfere with the renin-angiotensin-aldosterone system (ACE inhibitors or angiotensin II receptor antagonists). The chitotriosidase activity was determined by a fluorimetric method using 22 mmol/L 4-methylumbelliferyl b-D-N,N ,N -triacetylchitotrioside (Sigma Chemical Co.) in citrate-phosphate buffer, pH 5.2 [12]. Fluorescence was read on a Cary Eclipse fluorescence spectrometer (Agilent, Santa Clara, CA, USA). In the control group (n=243), the catalytic activity of CHIT was 6–162 nmol/mL/h.

Plasma ACE activity was measured by a spectrophotometric method utilising the substrate N-[3-(2-furyl)acryloyl]−L-phenylalanylglycylglycine (Trinity Biotech, St. Louis, SAD) on an Olympus AU 2700 automated analyser (Beckman Coulter Biomedical Ltd.). The normal range of ACE concentrations was 30–80 IU/L.

Since both serum chitotriosidase and ACE levels were not normally distributed (as revealed by theShapiro-Wilk test of normality), their values were logarithmically transformed to perform parametric statistical analysis (Pearson’s partial correlation, multiple regression and binomial logistic regression analysis), and results were presented as median values with interquartile ranges (IQR). Statistical analysis was performed using SPSS (IBM, Chicago, Illinois, USA).

## Results

The study included 90 patients with a mean age of 48.5±11.4 years. There were 31 (34.4%) males and 59 (65.6%) females. Baseline study characteristics are presented in Table 1. A total of 23 (25.6%) patients had Lofgren’s syndrome – an acute form of the disease that consists of mediastinal lymphadenopathy, erythema nodosum and arthritis. Taking into consideration clinical presentation, radiography and biomarkers (ACE and CHT), 53 (58.9%) patients had an active form of the disease (either active, chronic or reactivated in patients with previous remission). The majority of patients (50 (55.6%)) were on prednisone, with 13 (14.5%) of patients on other forms of treatment. A total of 44 (48.9%) patients had the second stage of sarcoidosis on radiography finding- lymphadenopathy and lung parenchymal disease.

**Table 1:** Baseline characteristics of patients. *Mean±Sd

		n (%)
Age, years*		48.5±11.4
Gender	male	31 (34.4)
	female	59 (65.6)
Acute form<br>(Lofgren syndrome)	yes	23 (25.6)
	no	67 (74.4)
Activity	active	53 (58.9)
	not active	37 (41.1)
Treatment	no treatment	27 (30)
	prednisone	50 (55.6)
	methotrexate	6 (6.7)
	prednisone+<br>methotrexate	6 (6.7)
	other treatment	1 (1.1)
Radiography stage	0	9 (10)
	1	9 (10)
	2	44 (48.9)
	3	2 (2.2)
	4	0 (0)

All patients had pulmonary form, of which 26 (28.9%) also had other localisations (cutaneous, neuro, cardiac, or other). Table 2 shows the symptoms the patients had at the initiation of the study.

**Table 2:** Symptoms at the beginning of the study.

Symptom	n (%)
Fatigue	64 (71.1)
Coughing	14 (15.6)
Chest pain	25 (27.8)
Dyspnoea	15 (16.7)
Pain in bones	56 (62.2)
Skin lesions	1 (1.1)
Headache	10 (11.1)
Diplopia	0 (0)
Cranial nerve affliction	0 (0)
Wrist oedema	16 (17.8)
Trouble sleeping	34 (37.8)

Changes in median values of biomarkers and SUVmax before and after the follow-up period are presented in Table 3. The median chitotriosidase level decreased significantly during the follow-up period (p=0.0045). The level of ACE did not change significantly (p=0.4850). SUVmax statistically considerably decreased during the follow-up (p<0.001).

**Table 3:** Changes in median values of biomarkers and SUVmax during the follow-up period. IQR – interquartile range (25^th^–75^th^ percentile); ACE – angiotensin-converting enzyme; CHT – chitotriosidase; SUVmax – maximum standardised uptake value.<br>* Wilcoxon signed-rank test; p<0.05 – statistically significant difference

	Baseline,<br>median (IQR)	After follow-up,<br>median (IQR)	p*
ACE, U/L	34 (23–53)	37 (23–53)	0.4850
CHT, nmol/mL/h	154.3 (65.8–224.1)	93.1 (46.8–163.5)	0.0045
SUVmax	7.0 (4.9–9.5)	3.5 (0.0–6.0)	<0.0001

Correlation analysis was performed between SUVmax values, ACE and chitotriosidase levels at the baseline and after the follow-up (Table 4). SUVmax and chitotriosidase levels were significantly correlated at the baseline and after the follow-up period, regardless of gender, age, duration of disease and radiography stage, while SUVmax and ACE levels were not. The correlation between ACE and chitotriosidase levels was significant only after the follow-up.

**Table 4:** Pearson’s correlation between SUVmax and biomarkers, after adjustment for gender, age, disease duration, radiography stage before and after the follow-up period. ACE – angiotensin-converting enzyme; CHT – chitotriosidase; SUVmax – maximum standardised uptake value; p<0.05 – statistically significant correlation.

	logACE, U/L	logCHT, nmol/mL/h
Baseline, Pearson’s partial r (p)		
logCHT, nmol/mL/h	-0.052 (0.632)	/
SUVmax	0.001 (0.993)	0.342 (0.001)
After follow-up, Pearson’s partial r (p)		
logCHT, nmol/mL/h	0.258 (0.016)	/
SUVmax	0.109 (0.319)	0.440 (<0.001)

Multiple regression was run to predict SUVmax before and after the follow-up period from ACE and chitotriosidase values, gender, age, duration of disease and radiography stage. Of these variables, only chitotriosidase statistically significantly added to the prediction of SUVmax both before and after the follow-up (Table 5).

**Table 5:** Multiple regression analysis of the predictive potential of the examined parameters on SUVmax before and after the follow-up. ACE – angiotensin-converting enzyme; CHT – chitotriosidase; SUVmax – maximum standardized uptake value; p<0.05 –statistically significant predictive value.

**Baseline, F(6, 83)=2.202, p=0.050, R^2^=0.137**		
Variable	B	p
Age	0.026	0.633
Gender	-1.017	0.384
Duration of the disease	-0.048	0.487
Radiography stage	-0.673	0.380
Log ACE	0.286	0.855
Log CHT	3.467	0.001
**After follow-up, F(6, 83)=4.119, p=0.001, R^2^=0.229**		
Variable	B	p
Age	0.008	0.860
Gender	-1.905	0.071
Duration of the disease	-0.027	0.659
Radiography stage	-0.112	0.863
Log ACE	-0.078	0.959
Log CHT	4.632	<0.001

A logistic regression was performed to determine the effects of ACE, chitotriosidase, age, gender, disease duration, and radiography stage on the likelihood that the SUVmax will be negative (≤1) before and after the follow-up period. The logistic regression model at the baseline was not statistically significant χ^2^(6)=3.457, p=0.750. The model explained only 12.4% (Nagelkerke R^2^) of the variance in SUVmax. On the other hand, the logistic regression model after the follow-up period, when the appropriate therapy protocol was applied, was statistically significant χ^2^(6)=20.495, p=0.002; the Hosmer and Lemeshow test of model fit indicated a good fit (p=0.182),i.e. there was no difference between the observed and predicted model. The model explained 27.7% (Nagelkerke R^2^) of the SUVmax variance, correctly classifying 70% of cases. If values of chitotriosidase were lower, it was 18.3 times more likely that the SUVmax would be negative (Table 6).

**Table 6:** Binomial logistic regression of the effects of examined parameters on the likelihood of negative SUVmax (SUVmax<1). ACE – angiotensin-converting enzyme; CHT – chitotriosidase; SUVmax – maximum standardised uptake value; p<0.05 – statistically significant predictive value.

**Baseline, χ^2^(6)=3.457, p=0.750, Nagelkerke R^2^=0.124**		
Variable	OR (95% CI)	p
Age	1.049 (0.928–1.186)	0.445
Gender	0.732 (0.056–9.618)	0.812
Duration of the disease	0.914 (0.800–1.045)	0.187
Radiography stage	1.620 (0.349–7.516)	0.538
Log ACE	0.163 (0.002–15.327)	0.434
Log CHT	1.633 (0.169–15.808)	0.672
**After follow-up, χ^2^(6)=20.495, p=0.002, Nagelkerke R^2^=0.277**		
Variable	OR (95% CI)	p
Age	1.000 (0.949–1.054)	0.997
Gender	1.617 (0.476–5.491)	0.441
Duration of the disease	1.012 (0.943–1.087)	0.732
Radiography stage	1.292 (0.613–2.724)	0.501
Log ACE	1.490 (0.249–8.900)	0.662
Log CHT	18.307 (3.694–90.713)	<0.001

**Figure 1:**
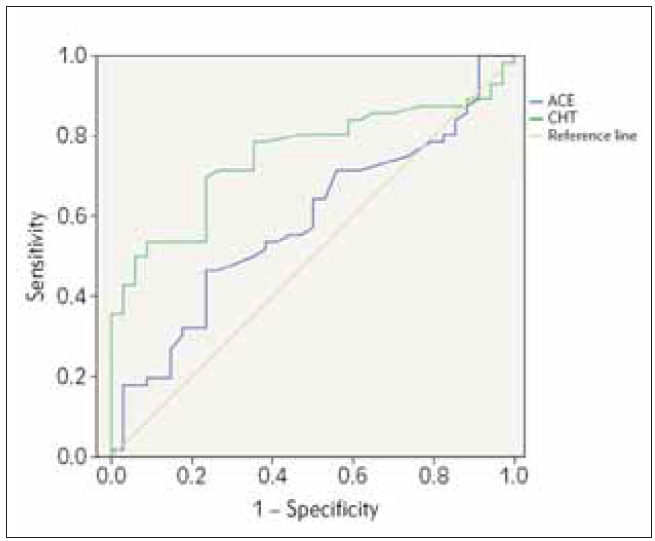
Receiver Operating Curves for prediction of negative SUVmax (SUVmax<1) of ACE and chitotriosidase.

ROC analysis showed an AUC of 0,751 (p < 0.001) for chitotriosidase value after the follow-up. The cut-off value of 47.4 nmol/mL/h had a sensitivity of 83.9% and a specificity of 42.2%. AUC for ACE was not statistically significant, showing its poor diagnostic ability for negative SUVmax. ROC analysis is presented in Figure 1 and Table 7.

**Table 7:** Area Under the Receiver Operating Curve (AUC) of ACE and CHT for prediction of negative SUVmax (SUVmax<1). ACE – angiotensin-converting enzyme; CHT – chitotriosidase; p<0.05 – a statistically significant difference from the AUC=0.5.

	Cut off, nmol/mL/h	Sensitivity %	Specificity %	AUC	95% CI	SE	p
ACE	29.5	64.3	50.0	0.584	0.464–0.703	0.061	0.184
CHT	47.4	83.9	42.2	0.751	0.651–0.851	0.051	<0.001

## Discussion

In this study, we tested the correlation between the levels of the established biomarkers in patients with histologically confirmed sarcoidosis, ACE and CHT and the level of SUVmax on PET/CT as an indicator of the change in the disease activity. The results demonstrated the significant predictive power of lower CHT values for the negative SUVmax values, which was not the case with ACE.

Positron emission tomography-computed tomo - graphy scan is a procedure to compare metabolic activity and radiological findings in many diseases. Fluor-18-Deoxyglucose Positron Emission Tomography (18F-FDG PET) has shown high sensitivity in detecting sarcoidosis and occult lesions [13] [14]. 18F-FDG PET, despite detecting occult lesions, can also help to detect the optimal lesion for biopsy [15]. The level of inflammation, measured as maximum standardised uptake value (SUVmax), detected on 18F-FDG PET, has been shown to predict the treatment outcome; the higher level of SUVmax has demonstrated a need for prolonged treatment [16]. 18F-FDG PET is a relatively safe method, although it does have certain limitations. Infections, malignancies and other inflammatory diseases can produce false positive findings; results in obese patients (BMI 30) and patients with non-regulated diabetes can be challenging to interpret due to elevated basal metabolism and dysregulation of glucose uptake, respectfully [17]. It should also be noted that 18F-FDG PET should not be utilised in patients who are pregnant or suspect of pregnancy. Another aspect to consider is the intervention’s financial aspect and the relative rarity of devices compared to X-ray and computer tomography.

The discovery of specific biomarkers should help in diagnostics and response to treatment [18]. Ideal biomarkers should be highly sensitive, specific for the exanimated disease, noninvasive and repeatable. Sarcoidosis, a heterogeneous disease with possible multiple organ involvement, brings another level of difficulty in determining the most optimal biomarker [19]. ACE and CHT are most commonly used as biomarkers in diagnosing and treating sarcoidosis.

ACE is a glycoprotein predominantly located in human lungs and kidneys, whose primary function is the conversion of angiotensin I to angiotensin II and inactivating bradykinin. Increased serum levels of ACE are related to macrophage activity in granulomatous diseases. They can be found not only in sarcoidosis but also in tuberculosis, silicosis, asbestosis, Gauchers disease, and leprosy, to name a few [20] [21]. Between 30–80% of sarcoidosis patients have increased ACE levels, sensitivity ranges between 22 and 86%, and specificity between 54 and 95% [22]. It should be noted that ACE inhibitors, used to treat arterial hypertension, lead to a decrease of ACE in serum and can lead to false negative findings. New research has shown a significant variation of ACE phenotypes in the general population, which also leads to variable levels of ACE in serum and can, therefore, mimic a granulomatous disease [23]. Due to the wide range of sensitivity and specificity and the influence of ACE inhibitors, the levels of ACE in diagnostics and response to treatment of sarcoidosis should be carefully analysed, as it can lead to false positive and negative results. Our results agree with these findings, showing low sensitivity and specificity (63.4% and 50%, respectively) of ACE for prediction of change of SUVmax in response to therapy.

CHT is an enzyme in active macrophages, belonging to a group of enzymes called chitinases, which partake in innate and adaptive immune systems. As being present in active macrophages, it is also noted to be elevated in granulomatous diseases, especially in sarcoidosis [1] [7]. Compared to ACE, chitotriosidase has shown a higher level of sensitivity and specificity: 86% sensitivity and 92.8% specificity [1] [7]. Chitotriosidase has also shown a certain level of correlation between its serum concentration and the radiological stage of sarcoidosis [24]. Just like ACE, CHT has shown increased levels in other diseases: Gaucher’s disease, malaria, multiple sclerosis, atherosclerosis, Alzheimer’s disease, and tuberculosis, although CHT has shown better results compared to ACE [25]. Our results showed significant predictive potential of CHT for decreasing SUVmax after six months of efficient therapy. If the CHT value was lower, it was 18.3 times more likely that the SUVmax would be negative. The cut-off value of 47.7 nmol/mL/h had a sensitivity of 83.9% with a rather low specificity, which may be explained by the wide range of diseases accompanied by CHT elevation.

To our knowledge, this is the first study dealing with the role of biomarkers in collaboration with PET/CT scans in monitoring disease activity and therapy. The main limitation of our study was a relatively small number of participants; however, sarcoidosis is considered a rare disease by Orphanet, and we have applied a relatively expensive method. Therefore, the replication of this study under similar criteria is not expected. For future studies, it might be useful to include more patients with extrapulmonary manifestations of the disease. Also, the effects of different therapy approaches would be possible to evaluate with the inclusion of more participants treated with different therapies.

## Conclusion

Previous studies have shown the importance of developing adequate biomarkers for sarcoidosis [26] [27]. 18F-FDG PET has high sensitivity, but it is not always possible to perform the scan in everyday practice, especially to repeat it to determine the efficiency of therapy. We have compared the two most commonly used biomarkers, ACE and CHT, with the results of 18F-FDG PET before and after the follow-up, and have shown that there is a strong positive correlation between the level of CHT and 18F-FDG PET finding and that there is no strong correlation between the level of ACE and 18F-FDG PET. CHT showed a significant predictive ability to decrease the sarcoidosis activity, which represented the decrease of SUVmax as the effect of therapy. However, it should be noted that the biomarker for sarcoidosis with high enough sensitivity, specificity, and stability in all forms of sarcoidosis has still not been discovered.

## Dodatak

### Funding

This research was funded by the Ministry of Education, Science and Technological Development of the Republic of Serbia (Project No. 200110).

### Conflict of interest statement

All the authors declare that they have no conflict of interest in this work.
